# Prevalence and outcome of abdominal vascular injury in severe trauma patients based on a TraumaRegister DGU international registry analysis

**DOI:** 10.1038/s41598-021-99635-9

**Published:** 2021-10-12

**Authors:** Mohammad Esmaeil Barbati, Frank Hildebrand, Hagen Andruszkow, Rolf Lefering, Michael J. Jacobs, Houman Jalaie, Alexander Gombert

**Affiliations:** 1 Department of Vascular and Endovascular Surgery, University Hospital Aachen, Aachen, Germany; 2 Department of Orthopedic Trauma, University Hospital Aachen, Aachen, Germany; 3 Institute for Research in Operative Medicine (IFOM), University Witten/Herdecke, Cologne, Germany

**Keywords:** Trauma, Prognosis, Vascular diseases, Peripheral vascular disease

## Abstract

This study details the etiology, frequency and effect of abdominal vascular injuries in patients after polytrauma based on a large registry of trauma patients. The impact of arterial, venous and mixed vascular injuries on patients’ outcome was of interest, as in particular the relevance of venous vessel injury may be underestimated and not adequately assessed in literature so far. All patients of TraumaRegister DGU with the following criteria were included: online documentation of european trauma centers, age 16–85 years, presence of abdominal vascular injury and Abbreviated Injury Scale (AIS) ≥ 3. Patients were divided in three groups of: arterial injury only, venous injury only, mixed arterial and venous injuries. Reporting in this study adheres to the STROBE criteria. A total of 2949 patients were included. All types of abdominal vessel injuries were more prevalent in patients with abdominal trauma followed by thoracic trauma. Rate of patients with shock upon admission were the same in patients with arterial injury alone (n = 606, 33%) and venous injury alone (n = 95, 32%). Venous trauma showed higher odds ratio for in-hospital mortality (OR: 1.48; 95% CI 1.10–1.98, p = 0.010). Abdominal arterial and venous injury in patients suffering from severe trauma were associated with a comparable rate of hemodynamic instability at the time of admission. 24 h as well as in-hospital mortality rate were similar in in patients with venous injury and arterial injury. Stable patients suspected of abdominal vascular injuries should be further investigated to exclude or localize the possible subtle venous injury.

## Introduction

Trauma-related abdominal vascular injuries are associated with a relevant mortality rate^1^. Even in the setting of a Primary Trauma Center and after prompt diagnosis, abdominal trauma involving major vessel injury remains challenging to treat^2^. Affected patients are very likely to require early and aggressive resuscitation measures in order to avoid or treat blood loss-associated acidosis, coagulopathy and hypothermia^3,4^. The relevance of aortic or iliac artery injury for patients’ outcome after blunt and penetrating abdominal trauma has been well described^5,6^. Blunt thoracic aortic injury is a common life-threatening complication of high velocity injury, which has gained attention within the last two decades as endovascular treatment options had improved patients’ outcome significantly^7^ In case of blunt abdominal aortic injury, literature is scarce and only few is known regarding outcome and optimal treatment strategy. Based on their manuscript, Shalhub et al. recommend conservative therapy in case of intimal tear and immediate emergency repair in case of rupture^8^. However, literature dealing with the impact of major venous vessel injury on patients’ outcome after polytrauma is scarce^9^. If available, the impact of abdominal major venous vessel injury is displayed in combination with aortic or iliac artery lesion^1^.

Outcome after isolated venous injury of all causes is directly related to a high mortality rate^10^. Using the Europe-wide data assessment of the TraumaRegister DGU we aimed to evaluate the impact of major abdominal vessel injury in severely injured polytrauma patients treated in primary trauma centers. Furthermore, the relevance of major venous vessel trauma and its impact on patients’ survival could be analyzed for the first time in a multicenter registry setting.

## Materials and methods

In the present study, data is retrospectively analyzed from the TraumaRegister DGU (TR-DGU). Regarding the manuscripts reporting we adhere to the STROBE guidelines^11^.

### Database

The TraumaRegister DGU of the German Trauma Society was founded in 1993. The aim of this multi-center database is a pseudonymised and standardized documentation of severely injured patients. Data is collected prospectively in four consecutive time phases from the site of the accident until discharge from hospital: (A) pre-hospital phase, (B) emergency room and initial surgery, (C) intensive care unit and (D) discharge. The documentation includes detailed information on demographics, injury pattern, comorbidities, pre- and in-hospital management, course on intensive care unit, relevant laboratory findings including data on transfusion and outcome of each individual. The inclusion criterion is admission to hospital via emergency room with subsequent ICU/ICM care or reach the hospital with vital signs and die before admission to ICU.

The infrastructure for documentation, data management, and data analysis is provided by AUC—Academy for Trauma Surgery (AUC—Akademie der Unfallchirurgie GmbH), a company affiliated to the German Trauma Society. The scientific leadership is provided by the Committee on Emergency Medicine, Intensive Care and Trauma Management (Sektion NIS) of the German Trauma Society (DGU). The participating hospitals submit their pseudonymised data to a central database via a web-based application. Scientific data analysis is approved according to a peer review procedure laid down in the publication guideline of TraumaRegister DGU. The participating hospitals are primarily located in Germany (90%), but a rising number of hospitals of other countries contribute data as well (Austria, Belgium, China, Finland, Luxembourg, Slovenia, Switzerland, The Netherlands, and the United Arabian Emirates). Currently, almost 30,000 cases from more than 650 hospitals are entered into the database annually. Participation in TraumaRegister DGU is voluntary. For hospitals associated with TraumaNetzwerk DGU, however, the entry of at least a basic data set is obligatory for reasons of quality assurance.

Data for the current research is obtained from TraumaRegister DGU (TR-DGU) as a sizeable cohort in the period between 2002 and 2017 and is in line with the publication guidelines of the TraumaRegister DGU (TR-DGU project ID 2018-027). The study was conducted in accordance with the Declaration of Helsinki Ethical Principles and Good Clinical Practices and approved by local ethics committee (Ethical Review Board of the University Hospital RWTH Aachen, Germany). The informed consent was waived by the [Ethical Review Board of the University Hospital RWTH Aachen, Germany] due to the retrospective nature of study.

### Patient groups and definitions

Patient selection was carried out according to the following criteria: (1) online documentation of European trauma centers since 2002, (2) age 16–85 years, (3) patients with serious injury (maximum Abbreviated Injury Scale ≥ 3). Early transferred out-patients (within 48 h after admission) were excluded in order to avoid double counting from both hospitals. Patients with vascular injuries in the abdomen were divided in three groups based on type of abdominal vessel injuries: arterial injury only (AI), venous injury only (VI), and mixed arterial and venous injuries (AVI). Patients without an abdominal vascular trauma served as control group. The Abbreviated Injury Scale (AIS) and Injury Severity Score (ISS) has been applied for injury grading^12^. The updated Revised Injury Severity Classification score (RISC II)^13^ was applied to adjust the observed mortality rates.

All participating hospitals are classified as supra-regional (level 1), regional (level 2) or local (level 3) trauma centers based on the availability of human and technical resources^14^. Organ failure was defined according to the Sequential Organ Failure Assessment (SOFA) where 3 or 4 points per organ was considered as organ failure. Multiple organ failure was defined as parallel failure of two or more organs for at least 2 days. Sepsis was defined according the ACCP/SCCM Consensus Conference (1992) as Systemic Inflammatory Response Syndrome (SIRS) plus a documented infection^15^.

As this is a retrospective study, there is a relevant risk for selection bias. Efforts were made to reduce the risk of relevant confounders; the data of the included patients has been initially assessed by a non-involved statistician and an independent committee assessed the reliability of the presented findings prior to publication.

### Statistical analysis

Descriptive analysis was presented as number of cases with percentage for categorical variables and mean with standard deviation (SD) for continuous measurements. No imputation was performed for missing data; all results refer to valid entries only. The decision not to use imputation in case of missing values is the usual standard in TR-DGU analyses since the amount of missing values in rather low (availability > 95% in the majority of variables. Furthermore, not all variables could be imputed adequately (if highly correlated other variables were missing) and selected checks (age, ISS, mortality) suggested that missing was at random in most instances. However, there is another feature of the TR-DGU which allows a hospital to participate with the standard version (about 100 data per case), or with a reduced version (about 40 data per case). This was introduced by our Trauma Society in order to limit the amount of work for documentation. Thus some data would not be available for ALL cases but only for those cases treated in hospitals which use the standard documentation. This has been described in the “Methods” section. Since larger hospitals more frequently use the standard data version we indicated findings available in the standard version only in the Tables where necessary. This has been done to indicate a careful interpretation (potential bias). The effect of vascular injury on outcome (hospital mortality) was evaluated with a logistic regression analysis. Other independent predictors in this analysis were the RISC II score (a combination of 15 predictive factors available on admission), massive transfusion, and hospital level of care. Results are presented as odds ratio (OR) with 95% confidence interval (95% CI). All analyses were performed using SPSS statistical software (version 24, IBM Inc., Armonk, NY, USA).

### Ethical approval and consent to participate

The present study is in line with the publication guidelines of the TraumaRegister DGU and is registered as TR-DGU Project ID 2018-027. As register data are assessed anonymously, individual informed consent is not required.

## Results

Table 1 summarizes the study group’s basic characteristics; abdominal vascular injury was present in 2949 patients (1.6% of all patients considered). Isolated arterial injury was observable in 83.4% (n = 2459). There were 383 patients (13%) admitted suffering from isolated venous injury and 107 patients (3.6%) had both arterial and venous injuries. Number of patients with ISS > 16 was 341 (89%) in patients with VI, 2236 (92%) in patients with AI, and 98 (92%) in patients with AVI. Blunt trauma was the most common mechanism responsible for abdominal vascular injuries in all three groups. Traffic injuries were the most common cause (n = 1720, 69.9%). Within the group of 230 assaulted patients, 179 (77.8%) sustained stabbing injuries and 51 (22.2%) patients had gunshot wounds. Out of the 2949 patients, 948 (32.1%) were hemodynamically unstable at the time of admission.

**Table 1:** Basic characteristics of 2949 patients with abdominal vascular injuries.

	Arterial injury only *n* = 2459	Venous injury only *n* = 383	Mixed arterial and venous injuries *n* = 107
Age (years)^a^	48.7 ± 19.1	44.0 ± 18.3	43.8 ± 17.6
Males	1831 (75%)	267 (70%)	83 (78%)
ISS ≥ 16	2236 (92%)	341 (89%)	98 (92%)
ISS^a^	33.8 ± 15.6	33.5 ± 16.5	35.9 ± 15.5
Penetrating trauma	230 (10%)	58 (16%)	29 (28%)
Prehospital shock (syst. BP ≤ 90 mmHg)	606 (33%)	95 (32%)	37 (47%)
Shock at ED admission (syst. BP ≤ 90 mmHg)	777 (35%)	125 (36%)	46 (47%)
**Mechanism of injury**			
Traffic accident—car/lorry	821 (34%)	120 (32%)	26 (24%)
Traffic accident—motorcycle	344 (14%)	58 (15%)	12 (11%)
Traffic accident—bicycle	96 (4%)	25 (7%)	5 (5%)
Traffic accident—pedestrian	167 (7%)	38 (10%)	8 (8%)
High fall (> 3 m)	438 (18%)	47 (13%)	15 (14%)
Low fall (< 3 m)	143 (6%)	14 (4%)	3 (3%)
Gunshot	28 (1%)	13 (3%)	10 (9%)
Stabbing	140 (6%)	28 (7%)	11 (10%)

*ISS *Injury Severity Score.

^a^Mean with standard deviation.

The distribution of arterial and venous injuries in patients with severe injuries (AIS ≥ 3) of head, thoracic, abdomen and extremities are shown in Table 2. All types of vessel injuries were more prevalent in patients with severe abdominal trauma followed by severe thoracic trauma as the second most common cause.

**Table 2:** Distribution of arterial and venous vessel injuries in patients with relevant injury (AIS ≥ 3).

Affected vessel	Relevant head trauma *n* = 84,544	Relevant thoracic trauma *n* = 91,437	Relevant abdominal trauma *n* = 25,426	Relevant injury of the extremities *n* = 58,555
Abdominal vascular injury	825 (0.9%)	1980 (2.1%)	3425 (13.4%)	1640 (2.8%)
Abdominal aortic or arterial injury	589 (0.7%)	1431 (1.6%)	2566 (10.1%)	1173 (2.0%)
Abdominal venous injuries	117 (0.1%)	271 (0.3%)	466 (1.8%)	229 (0.4%)
Inferior vena cava	48 (< 0.1%)	107 (0.1%)	153 (0.6%)	67 (0.1%)
Iliac vein	26 (< 0.1%)	42 (< 0.1%)	87 (0.3%)	84 (0.1%)
Other abdominal veins	45 (< 0.1%)	129 (0.1%)	153 (0.6%)	87 (0.1%)

Patients with higher abdominal AIS score were more likely to be hemodynamically unstable and required blood transfusion more frequently. In this subgroup of patients, rate of cessation of the trauma resuscitation algorithm and need of emergency surgery was higher with increasing abdominal AIS (Table 3).

**Table 3:** Early clinical management in subgroups according to severity of abdominal vascular trauma.

	Controls	AIS-3	AIS-4	AIS-5
No. of patients	184,276	1380	1323	211
Systolic BP ≤ 90 mmHg on admission	16,399 (10%)	316 (25%)	500 (42%)	120 (64%)
Blood transfusion	22,202 (12%)	530 (39%)	717 (56%)	133 (66%)
Massive transfusion	3831 (2%)	185 (14%)	282 (22%)	56 (28%)
Number of pRBC	0.7 ± 3.1 M: 0	3.8 ± 8.1 M: 0	6.3 ± 10.8 M: 2	8.0 ± 12.2 M: 4
Emergency surgery 1	2377 (3%)	87 (13%)	132 (21%)	24 (24%)
Emergency surgery 2	40,905 (26%)	605 (51%)	751 (64%)	132 (71%)
WB-MSCT	127,583 (70%)	1094 (80%)	974 (75%)	124 (60%)

Total numbers and percentages of each group are given with the total number of available datasets for each characteristic in parenthesis.

Total patient numbers may vary for each procedure and characteristic because of incomplete data transmission or transmission of basic datasets. Basic datasets do not include information on emergency/early surgery.

Massive transfusion: ≥ 10 units of packed red blood cells.

Emergency surgery 1: immediate surgery requiring cessation of the implemented trauma resuscitation algorithm (2002 until 2015); Emergency surgery 2: Intervention (since 2009, from a list of 7 critical interventions, in the ER or directly consecutive).

*BP* blood pressure, *pRBC* packed red blood cells, *M* median, *WB-MSCT* whole-body multi-slice computed tomography.

Patients with VI or AVI showed slightly higher mortality rates within the first 24 h as well as increased in-hospital mortality rates if compared with the control group (Table 4).

**Table 4:** Impact of venous injury on patient outcomes after abdominal trauma.

	Only arterial injury *n* = 2459	Only venous injury *n* = 383	Both arterial and venous injuries *n* = 107
Mortality in first 24 h	448 (18.2%)	83 (21.7%)	35 (32.7%)
Hospital mortality	689 (28.0%)	127 (33.2%)	47 (43.9%)
Multiple organ failure^a^	648 (47%)	114 (51%)	37 (65%)
Sepsis^a^	202 (15%)	31 (14%)	16 (28%)
Kidney failure^a^	223 (16%)	34 (15%)	18 (32%)
Days of mechanical ventilation	6.8 ± 12.2 M: 1	6.5 ± 11.4 M: 1	9.2 ± 19.2 M: 1.5
ICU length of stay (day)	11.6 ± 15.9 M: 5	11.1 ± 15.8 M: 5	16.3 ± 29.7 M: 3
Hospital length of stay (day)	24 ± 26 M: 16	21 ± 23 M: 15	27 ± 38 M: 11
Blood transfusion	1120 (46%)	201 (53%)	81 (77%)
Massive transfusion (≥ 10 units of pRBC)	396 (16%)	86 (23%)	46 (44%)
FFP transfusion	766 (32%)	144 (38%)	67 (64%)
Average number of pRBC	4.7 ± 9.1 M: 0	6.3 ± 10.6 M: 2	13.0 ± 16.1 M: 8

Continuous variables presented with mean, SD, and median.

*pRBC* packed red blood cells, *FFP* fresh frozen plasma, *M* median.

^a^Available only in patients with standard documentation (68% of all cases).

Moreover, the rate of multiple organ failure, sepsis and acute kidney failure was higher in patients with VI or AVI. The mean hospital length of stay in patients suffering from AI, VI and AVI were 24, 21 and 27 days, respectively (Table 5).

**Table 5:** Results of logistic regression analysis with in-hospital mortality as dependent variable.

Predictor	Coefficient (SE)	p-value	Odds ratio (OR)	95% confidence interval of OR
RISC II score	− 0.96	< 0.001	0.383	0.379–0.388
**Hospital level of care**^**a**^				
Regional trauma center (level 2)	0.04	0.094	1.04	0.99–1.10
Local trauma center (level 3)	0.02	0.620	1.02	0.93–1.12
Arterial injury in the abdomen^b^	0.43	< 0.001	1.54	1.35–1.77
Venous injury in the abdomen^b^	0.57	< 0.001	1.77	1.33–2.36
Constant term	− 0.05	0.001		

The analysis is based on 164,370 patients; Nagelkerke’s R^2^ = 0.585.

^a^Reference group: supra regional trauma center (level 1).

^b^Reference group: no such injury documented.

Blood transfusion (BT)and fresh-frozen plasma (FFP) transfusion rate as well as rate of massive transfusion (MT) were higher in patients with VI compared to AI (BT: 201 (53%) vs. 1120 (46%), FFP: 144 (38%) vs. 766 (32%) and MT: 86 (23%) vs. 396 (16%)) (Table 4).

A multivariable logistic regression model was calculated to evaluate the potential impact of abdominal vascular injury on mortality. Further independent predictors were the RISC II score, massive transfusion, and hospital level of care. In this analysis, isolated VI and isolated AI were significantly related to an increasedin-hospital mortality rate. Venous trauma showed higher odds ratio for in-hospital mortality if compared with AI (isolate AI: OR: 1.31; 95% CI 1.14–1.50, p < 0.001; isolated VI: OR: 1.48; 95% CI 1.10–1.98, p = 0.010) (Table 5).

## Discussion

The mortality rate of severely injured persons is negatively influenced by the presence of a hemorrhagic shock which is often caused by severe abdominal and pelvic trauma.

According to our data, road accidents account for most of the abdominal vascular injuries, followed by fall from heights as the second most important cause. Results from preceding studies indicated blunt trauma especially following road traffic accident is the main mechanism of abdominal vascular injuries in trauma patients^16,17^. AIs are more common in this setting, yet VIs are also likely to occur (Table 2) and should not be underestimated^18^. Inferior vena cava (IVC) injuries lead to high rates of morbidity and mortality. Studies reported that more than one-third of patients with an IVC injury has a mortality rate of more than 60% after admission to hospital^19,20^. Accordingly, hemodynamic status and prompt identification of bleeding source are in focus when treating patients suffering from abdominal vascular injuries^1^. In the vast majority of cases, intraabdominal hemorrhage may lead to metabolic acidosis followed by coagulopathy and hypothermia, the so-called lethal triad of trauma^21,22^.

In terms of the diagnosis of abdominal vascular injuries, preoperative assessment of hemodynamically unstable patients may include Focused assessment with sonography for trauma (FAST) or diagnostic peritoneal lavage to confirm the hemoperitoneum^23,24^. However, retroperitoneal injuries have no or just a small volume of free blood. Significant retroperitoneal VIs, such as those affecting the retrohepatic IVC, can be subtle, with patients presenting with no symptoms at all, or even with intermittent hypotension that reacts to resuscitation at the beginning. Asensio et al. reported 275 retroperitoneal hematoma in 302 patients with abdominal vessel injuries leading to an incidence of 91%^1^. Concerning the rapid diagnosis of retroperitoneal injuries it is advocated that even in stable patients suspected of abdominal vascular injuries, a triple-contrast abdominal CT scanning may be beneficial to localize the retroperitoneal vascular injuries and evaluate the extension of vessel involvement^25,26^.

Although the rate of hemodynamic instability at the time of admission was the same in patients with VI (36%) comparing to patients with AI (35%) in the current study, the rate of adverse outcome was significantly higher in patient with VI. Based on the present data, an increase in mass transfusion and multiple organ failure correlated significantly with involvement of VIs in patient suffering from abdominal vascular injuries. Consecutively, an increase in mortality rates within the first 24 h and during the hospital stay can be assessed after VI or AVI (Tables 1 and 4).

In the present study, the overall mortality rate was 29.2%, emphasizing a high mortality rate related to abdominal vascular injuries as reported in other studies (17–54%)^1,17,27–30^. Numerous factors predicting mortality in abdominal vascular injuries have been described before, i.e., the presence of shock, hypothermia, acidosis, arrhythmias, transfusion requirement, and number of injured vessels^28,30–33^. Our findings, after adjusting the mortality rate for covariates, showed that abdominal VI is associated with a significantly increased odds ofmortality (OR: 1.48; 95% CI 1.10–1.98, p = 0.010). This effect was than the adjusted odds of abdominal AI (OR: 1.31; 95% CI 1.14–1.50, p < 0.001). Correspondingly, the mortality rates in patients with VI was higher if compared with AI (21.7% vs. 18.2% in first 24 h, 33.2% vs. 28% in hospital mortality, respectively) (Table 4).

Based on our findings which were able to underline pre-existing results of smaller studies, efforts should be made to initiate a large, national or international registry focusing on prospective assessment of major vessel injury in high-velocity trauma. This could enable more reliable findings, helping to establish diagnostic and treatment guidelines for major vessel injuries.

The shortcomings of the study are similar to other studies using large registry databases. TR-DGU's initial aim was to register severely injured patients or those with multiple injuries and solely require ICU admission. It only includes in-hospital trauma fatalities, excluding victims that died at scene or during transport.

We were not able to analyze technical details of endovascular and open surgery. Moreover a separate analysis of the different venous segments was not possible because of the Trauma Register’s data collection. A further limitation is the lack of possibility to separate venous vessel injury from general mortality rate. Moreover, further risk factors and existing comorbidities were not available either. These facts reduce the validity of the presented information, as influencing factors could not be taken into account. Lack of follow-up outcomes for the included patient variables may have impacted the findings of this study.

## Conclusion

Abdominal VI are present in more than 25% of all patients with abdominal vascular injury in case of severe trauma. Patients suffering from VI showed a comparable and even slightly decrease in survival rate after hospital admission if compared to abdominal arterial vessel. Stable patients suspected for abdominal vascular injuries should undergo further scanning to investigate and localize the possible subtle VI.

## Data Availability

The datasets used and/or analyzed during the current study are available from the corresponding author on reasonable request.
